# Artificial intelligence approach for recommendation of pupil dilation test using medical interview and basic ophthalmologic examinations

**DOI:** 10.3389/fmed.2022.967710

**Published:** 2022-09-13

**Authors:** Hyunmin Ahn, Ikhyun Jun, Kyoung Yul Seo, Eung Kweon Kim, Tae-im Kim

**Affiliations:** ^1^Department of Ophthalmology, Yonsei University College of Medicine, Seoul, South Korea; ^2^Corneal Dystrophy Research Institute, Yonsei University College of Medicine, Seoul, South Korea; ^3^Saevit Eye Hospital, Goyang, South Korea

**Keywords:** artificial intelligence, machine learning, medical interview, ophthalmologic examination, pupil dilation test

## Abstract

**Purpose:**

To evaluate the value of artificial intelligence (AI) for recommendation of pupil dilation test using medical interview and basic ophthalmologic examinations.

**Design:**

Retrospective, cross-sectional study.

**Subjects:**

Medical records of 56,811 patients who visited our outpatient clinic for the first time between 2017 and 2020 were included in the training dataset. Patients who visited the clinic in 2021 were included in the test dataset. Among these, 3,885 asymptomatic patients, including eye check-up patients, were initially included in test dataset I. Subsequently, 14,199 symptomatic patients who visited the clinic in 2021 were included in test dataset II.

**Methods:**

All patients underwent a medical interview and basic ophthalmologic examinations such as uncorrected distance visual acuity, corrected distance visual acuity, non-contact tonometry, auto-keratometry, slit-lamp examination, dilated pupil test, and fundus examination. A clinically significant lesion in the lens, vitreous, and fundus was defined by subspecialists, and the need for a pupil dilation test was determined when the participants had one or more clinically significant lesions in any eye. Input variables of AI consisted of a medical interview and basic ophthalmologic examinations, and the AI was evaluated with predictive performance for the need of a pupil dilation test.

**Main outcome measures:**

Accuracy, sensitivity, specificity, and positive predictive value.

**Results:**

Clinically significant lesions were present in 26.5 and 59.1% of patients in test datasets I and II, respectively. In test dataset I, the model performances were as follows: accuracy, 0.908 (95% confidence interval (CI): 0.880–0.936); sensitivity, 0.757 (95% CI: 0.713–0.801); specificity, 0.962 (95% CI: 0.947–0.977); positive predictive value, 0.878 (95% CI: 0.834–0.922); and F1 score, 0.813. In test dataset II, the model had an accuracy of 0.949 (95% CI: 0.934–0.964), a sensitivity of 0.942 (95% CI: 0.928–956), a specificity of 0.960 (95% CI: 0.927–0.993), a positive predictive value of 0.971 (95% CI: 0.957–0.985), and a F1 score of 0.956.

**Conclusion:**

The AI model performing a medical interview and basic ophthalmologic examinations to determine the need for a pupil dilation test had good sensitivity and specificity for symptomatic patients, although there was a limitation in identifying asymptomatic patients.

## Introduction

**Figure 1:**
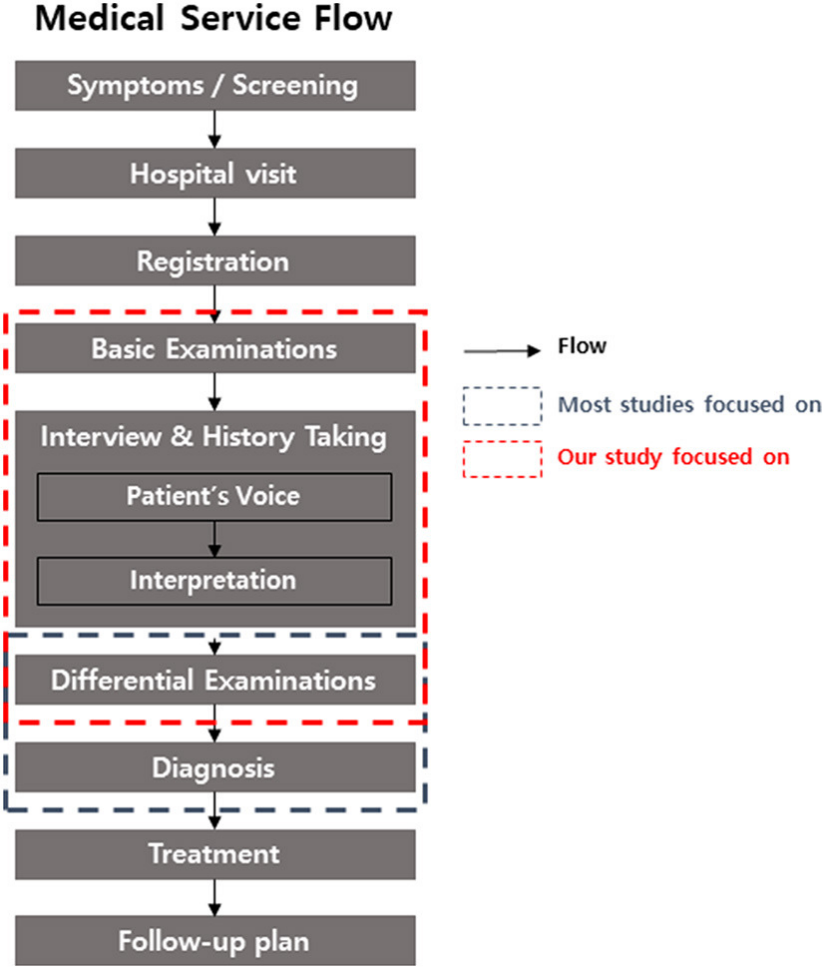
The flow of medical services from to doctors in this study.

In today's era, artificial intelligence (AI) is one of the hottest topics in all fields worldwide. Digital device, marketing, education, and AI itself are the target of AI development (1–4). The medical field also cannot escape from this trend. However, only very specific settings in clinical practice, such as the detection of arterial fibrillation, epilepsy seizure, and hypoglycemia, or the diagnosis of disease based on histopathological examination or medical imaging benefit from the application of medical AI (5). Recent research in ophthalmology showed that AIs with deep learning algorithms had an acceptable performance in ophthalmic imaging data, such as fundus photography and topography (6). However, there are various challenges in the application of AI in actual clinical practice, even with AI with good performance for imaging analysis (7). Considering the flow of medical services from patients to doctors (Figure 1), tremendous applications of AI are possible.

Patient visit time for outpatient clinic is one of the key issues to address in order to improve not only the quality of medical services but also the clinic efficiency (8). Minimizing the medical process reduces the patient's waiting time and medical costs while improving the satisfaction of service providers and beneficiaries. Pupil dilation test and fundus examination is performed to differentiate between intraocular diseases. The majority of anterior segment diseases are diagnosed using slit-lamp biomicroscopic examination. In contrast, diseases in the lens, optic nerve, vitreous, and chorio-retina are basically diagnosed using pupil dilation test (9). However, after pupil dilation, some important examinations such as near vision test, pupillary light reflex, and visual field examination have a limitation or bias. Moreover, considering the dilation time after discontinuing mydriatics, fundus examination is a turning point in the process of medical service in ophthalmology, from visit to treatment (10). Pupil dilation test can be performed for a patient with symptoms and signs that suggest the possibility of an intraocular disease after a medical interview and basic ophthalmologic examinations. However, in many cases, because these processes have a limitation to presume some intraocular diseases, this test is performed after an additional process that an ophthalmologist conducts directly, such as slit-lamp biomicroscopy. Moreover, many intraocular diseases are asymptomatic and are detected incidentally (11–13).

We considered using AI to simplify the medical service process through the automatic determination of pupil dilation test. There is no study on AI that recommends pupil dilation test. This study aimed to determine whether AI can recommend pupil dilation test appropriately when only basic ophthalmologic information is provided, as in our clinical situation.

## Materials and methods

The study was conducted in accordance with the tenets of the Declaration of Helsinki, and ethical approval for each follow-up was obtained from the institutional review board (IRB) of Yonsei University College of Medicine. All participants for prospective validation provided written informed consent before participating. For retrospective data, patient consent was waived after IRB approval (Protocol number 4-2022-0326).

### Participants

The study was conducted at Severance Hospital, Yonsei University College of Medicine, Republic of Korea. Medical records from 2017 to 2021 were analyzed. All patients who visited the outpatient clinic of Severance Eye hospital for the first time were included the study. The medical service process of the first visiting outpatient is presented in the flowchart in Figure 1. The key inclusion criteria were as follows: (1) completed medical interview and basic ophthalmologic examinations such as slit-lamp biomicroscopy, pupil dilation test, and fundus examination, (2) communicated directly (for children, including parents), and (3) medical records confirmed by a subspecialist. Patients who did not complete all examinations were excluded. The diagnosis, treatment, and follow-up plan were confirmed to exclude unspecified disease.

### Basic protocol for first-visiting patients

#### Medical interview and basic ophthalmologic examinations

Patients who visited the outpatient clinic for the first time were interviewed by ophthalmologists and experienced paramedics who had been trained for at least 2 years with confirmed hospital protocols. In the interview, chief complaint, comorbid symptoms, duration, systemic/ophthalmic history, and familial history were collected (see also Supplementary material 1). Systemic and ophthalmic diseases were categorized by the International Statistical Classification of Diseases and Related Health Problems, tenth revision (ICD-10) classification. After the medical interview, all patients underwent basic ophthalmologic examinations such as uncorrected distance visual acuity, corrected distance visual acuity if wearing glasses and contact lenses, autokeratometry, corrected distance visual acuity with autokeratometry, and intraocular pressure (IOP) with non-contact tonometry. When the IOP was under 7 mmHg or over 21 mmHg, the measurements were repeated twice. When the initial measurement of refraction or keratometric power by autokeratometry failed, a repeat measurement was performed.

#### Pupil dilation test and fundus examination

All new patients who visited our clinic were required to undergo a pupil dilation test and fundus examination.

#### Further processes

After medical interview and basic ophthalmologic examination, all new patients underwent additional examinations, or treatments after referral to subspecialist. All the contents of the medical processes were saved in the electronic medical record.

### AI modeling

**Figure 2:**
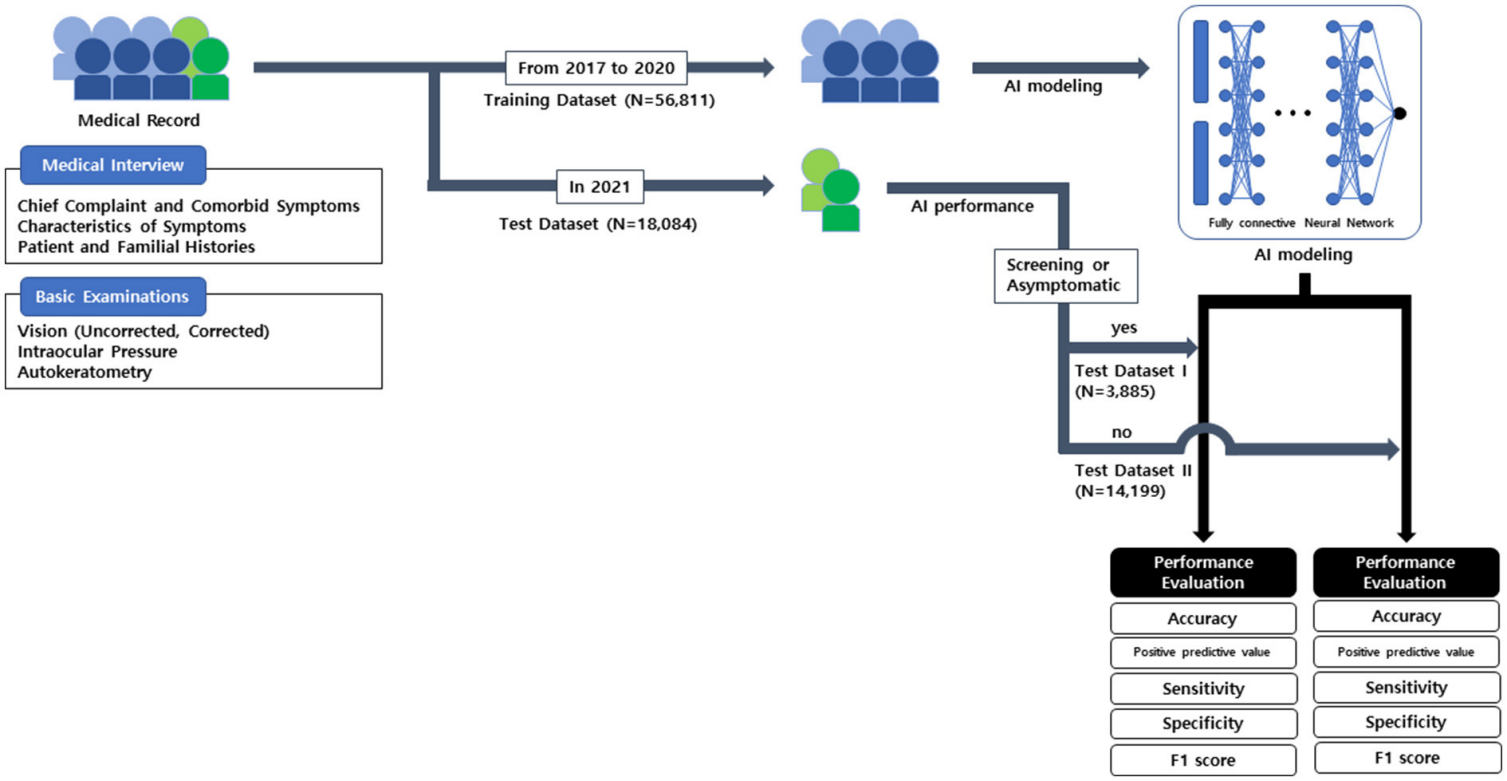
The process used in this study and artificial intelligence modeling for recommending pupil dilation test.

The overall process of AI modeling is described in Figure 2. AI modeling was constructed based on the electronic medical record by Python 3.8 program.

#### Training dataset

Prior to AI modeling, patients who first visited the outpatient clinic between 2017 and 2020 were included in the training dataset.

#### Test dataset

Patients who first visited the clinic in 2021 were included in the test dataset. First, patients who underwent an eye screening test for their systemic disease and treatment, as well as asymptomatic patients including consultation cases from other medical parts, were included in test dataset I. Subsequently, test patients that were not included in test dataset I, were included in test dataset II.

#### Input variables

The input variables were as follows: **(**1) General patient information, including age, sex, systemic/ophthalmologic history, and family history, (2) symptoms and events, and (3) results of the basic ophthalmologic examination (see also Supplementary material 1). Patients' symptoms were sorted based on the list in the website of American Academy of Ophthalmology and our previous study (14, 15). Characteristics, duration, time aspect, related events, and other purposes of visiting (i.e., health check-up and screening ophthalmic complications of systemic diseases and treatments) were also interpreted (see also Supplementary material 1). For model training, training dataset was split into training and validation data in a 3: 7 ratios. Standard scaler was used for visual acuities, IOP, and the values of autokeratometry.

#### Output variables

The output variable was set to the binary value of the need for a pupil dilation test (yes or no). The need for a pupil dilation test was determined when there were clinically significant lesions in any of the eyes. A clinically significant lesion for the pupil dilation test was defined as a lesion in the lens, vitreous, retina, and optic disc area. It was confirmed by each subspecialist when **one** or more of the following criteria were met: (1) clearly explained patient's subjective symptoms, (2) required additional follow-up with the possibility of exacerbation of disease and/or intervention within 3 months, and (3) required additional detailed examinations for treatment plan (see also Supplementary material 2). Lesions such as asymptomatic mild macular drusen, lattice degeneration without retinal break or vitreous traction, simple retinal pigmentation and chronic scars, low-risk glaucoma suspect with long-term follow-up over 6 months, and non-vision impairing cataract were not deemed clinically significant by subspecialists (16–19). Functional disorders and extraocular disorders that did not require a pupil dilation test were also not deemed clinically significant.

#### Model construction

AI modeling was conducted with fully connected deep neural network. The activation function for hidden layers was rectified linear (ReLU) function. Adam optimization was used. Accuracy was used as a metric. The depth of the hidden layer and the nodes in each hidden layer were automatically modulated with network topology. Batch and epoch size were automatically modulated. Dropout 0.5 and L2 regularization were used to prevent overfitting. Performance and loss were surveilled to prevent underfitting. The early stopping method was used.

### Statistical analysis

Statistical analysis was conducted with Python 3.8 program. Model performance was evaluated with accuracy, sensitivity (also called recall), specificity, and positive predictive value (also called precision); 95% confidence intervals (CIs) were used. The false-positive and false-negative cases in each test dataset were descriptively analyzed with the location of the lesion based on the ICD-10 classification. If the locations overlapped, all locations were considered.

## Results

In the training dataset, 56,811 patients were enrolled, with women accounting for 54.1%. The mean age of the patients was 57.5 ± 18.9 years. The clinically significant lesions for pupil dilation test were present in 65.1% of the patients. Of the clinically significant lesions, 28.9% were in the lens, 7.8% in the vitreous, 38.6% in the macular area, 12.6% in the peripheral retina, and 20.1% in the optic disc. A total of 3,885 patients were enrolled in test dataset I, and 14,199 patients were enrolled in test dataset II. The clinically significant lesions were present in 26.5% of patients in test dataset I and 59.1% of patients in test dataset II (Table 1).

**Table 1:** Characteristics of study patients in training dataset and test dataset I and II.

**Characteristics**	**Training (*N* = 56,811)**	**Test I (*N* = 3,885)**	**Test II (*N* = 14,199)**
Age (years, mean±SD)	57.5 ± 18.9	48.5 ± 22.3	60.0 ± 17.8
Sex (proportion of female, %)	54.1	60.2	54.2
Uncorrected distance visual acuity (logMAR, mean±SD)	0.57 ± 0.51	0.48 ± 0.52	0.57 ± 0.50
Corrected distance visual acuity (logMAR, mean±SD)^†^	0.19 ± 0.27	0.07 ± 0.08	0.19 ± 0.29
Intraocular pressure (mmHg, mean±SD)^‡^	15.3 ± 3.5	14.7 ± 3.4	15.2 ± 3.3
Spherical equivalent (diopters, mean±SD)^†^	−1.65 ± 3.06	−1.68 ± 2.81	−1.60 ± 3.45
Corneal power (diopters, mean±SD)^†^	43.33 ± 2.02	42.19 ± 3.75	43.30 ± 2.02
**Clinically significant lesion (% of eyes)**	65.1	26.5	59.1
Lens (% of clinically significant lesion)	28.9	35.3	23.5
Vitreous (% of clinically significant lesion)	7.8	2.5	7.0
Macula (% of clinically significant lesion)	38.6	14.8	40.5
Peripheral retina (% of clinically significant lesion)	12.6	30.0	15.3
Optic disc (% of clinically significant lesion)	20.1	25.5	19.7

† Measured by auto-keratometry.

‡ Measured by non-contact tonometry.

In test datasets I and II, the AI recommendation for pupil dilation test had an accuracy of 0.908 (95% CI: 0.880–0.936) and 0.949 (95% CI: 0.934–0.964), respectively. The sensitivity, specificity, and positive predictive value in test dataset I were 0.757 (95% CI: 0.713–0.801), 0.962 (95% CI: 0.947–0.977), and 0.878 (95% CI: 0.834–0.922), respectively, and those in test dataset II were 0.942 (95% CI: 0.928–0.956), 0.960 (95% CI: 0.927–0.993), and 0.971 (95% CI: 0.957–0.985), respectively (Table 2). F1 score was 0.813 in test dataset I and 0.956 in test dataset II.

**Table 2:** The performance of AI for recommendation pupil dilation test in test dataset I and II.

	**Test dataset I (*****n*** = **3,885)**		**Test dataset II (*****n*** = **14,199)**	
	**Estimate**	**95% CI**	**Estimate**	**95% CI**
Accuracy	0.908	0.880–0.936	0.949	0.934–0.964
Sensitivity (Recall)	0.757	0.713–0.801	0.942	0.928–0.956
Specificity	0.962	0.947–0.977	0.960	0.927–0.993
Positive predictive value (Precision)	0.878	0.834–0.922	0.971	0.957–0.985
F1 score	0.813	-	0.956	-

Table 3 shows the proportion of the locations of the clinically significant lesions in the false-negative and false-positive categories in the entire test dataset. In the false-negative category, 37% of the lesions were in the macular area, 28.1% in the optic disc, 20.1% in the peripheral retina, 10.3% in the lens, and 5.3% in the vitreous. In the false-positive category, 73% of the lesions were in the anterior segment, including the cornea and anterior chamber, and 10.7% were in the eyelid and extra-orbital area. Further, 17% of the false-positive cases had non-ophthalmologic causes.

**Table 3:** Locations of clinically significant lesions in false-negative and false-positive categories with overall test dataset.

**Locations**	**Proportions (%)**
**False-negative**	
Lens	10.3
Vitreous	5.3
Macula	37.2
Peripheral retina	20.1
Optic disc	28.1
**False-positive**	
Cornea and Anterior chamber	73.1%
Eyelid and Extra-orbital area	10.7%
Non-ophthalmologic	17.3%

## Discussion

The performance of the AI in recommending pupil dilation test using a medical interview and basic ophthalmologic examinations was good, with ~95% accuracy in symptomatic patients. However the AI had a limitation in detecting asymptomatic lesions in the lens, vitreous, chorio-retina, and optic nerve, and the sensitivity and positive predictive value of test dataset I was ~76 and 88%.

In the ophthalmologic service process, examination of vision and IOP are generally recommended at a visiting eye clinic (10). Ophthalmologists select additional differential and detailed examinations based on the information obtained from a medical interview and basic ophthalmologic examinations. AI automation is expected to improve the efficiency of the medical process (20). We aimed to develop a decision-making AI for ophthalmologic examinations as a type of AI that will help to reduce the time and cost of medical services.

Because the characteristics of study population in test dataset II were more similar to those in training dataset than in test dataset I, this AI model might perform better in test dataset II than in test dataset I. This study was conducted at a tertiary medical service institution, and the number of asymptomatic patients was smaller than the number of symptomatic patients as confirmed by the sample size of test dataset I which was smaller than the sample size of test dataset II. Analyzing AI model performance in detail, test dataset I had lower sensitivity and positive predictive value, which was attributed to the lower true positive ratio. According to previous studies, fundus examination is important to detect asymptomatic diseases (16). Glaucoma, diabetic retinopathy, and age-related macular degeneration are well-known diseases that are asymptomatic in the early phase (21–23). Ocular symptoms are not the only reason for a pupil dilation test; the patient's ophthalmologic history, systemic disease, and familial history are also considered (24–27). The importance of pupil dilation test and fundus examination in asymptomatic patients is a contrary evidence that the investigator cannot predict the disease of the posterior segment of the eye from symptoms alone. Moreover, the past history and familial history of the patient may be unclear or unrevealed. These problems were also reflected in the results of our study, especially in asymptomatic patients.

In this study, information obtained from the medical interview and basic ophthalmologic examinations used as the input dataset were limited in determining whether to conduct a pupil dilation test. The performance of the AI can be improved by changing the AI model or using a large sample size (28). We used several methods to overcome the technical problem. First, the hyperparameters, especially the number of hidden layers, nodes, batch size, and epochs were modulated automatically with surveillance of overfitting and underfitting. Increasing the number of hyperparameters does not always increase the performance of AI (29, 30). In this study, because there was no continuous performance improvement with the additional training process, and the plateau phenomenon was detected in all of the sequences with hyperparameter modulation, the possibility of underfitting was carefully estimated to be minimized. Second, we evaluated the performance with two validation datasets. We tried to determine whether the lower performance was due to a technical issue or a limitation of clinical factor. The results of this study suggest that insufficient performance in test dataset I of the AI model was caused by asymptomatic lesions, limitation of clinical factor, and aleatoric uncertainty. In order to improve the performance of the AI in cases with asymptomatic lesions, completely new input information is needed rather than simply increasing the sample size or changing the AI model.

This study has some limitations. First, the dataset was collected from a tertiary care hospital. The proportion of patients with clinically significant lesions in tertiary care hospitals is different from that in a primary care service. The performance of AI may vary in a primary care setting, depending on the application area, such as telemedicine. Prospective applicable research in various clinical settings is needed. Second, the result of this study is applicable only for patients visiting the clinic for the time. AI in patients with previous visiting history is different, and it could be considered with other AI models such as recurrent neural network. Third, the patients' symptoms were interpreted by medical personnel and did not directly reflect the patients' expression. This study did not evaluate the use of AI by patients. An advanced AI using dataset directly expressed by patients in ways such as speech or writing is now being planned. Finally, the definition of “need for pupil dilation test” was determined by each subspecialist in our hospital. The definition might be clinically acceptable and the controversial cases between subspecialists which were <0.1% in this study were excluded in this study. However, bias from individual cases could not be completely excluded. Perhaps this issue depends on the protocol guidelines within hospital or group.

In conclusion, the AI recommending pupil dilation test had a good performance with only basic ophthalmologic information for symptomatic lesions, although there was a limitation of the performance for asymptomatic lesions.

## Data availability statement

The raw data supporting the conclusions of this article will be made available by the authors, without undue reservation.

## Ethics statement

The studies involving human participants were reviewed and approved by the Institutional Review Board (IRB) of Yonsei University College of Medicine. Written informed consent from the participants' legal guardian/next of kin was not required to participate in this study in accordance with the national legislation and the institutional requirements.

## Author contributions

HA and T-iK designed the study and wrote the original draft of the manuscript. HA collected patient data, performed the artificial intelligence modeling, and analysis. IJ, KS, and EK are responsible for reviewing the manuscript. All authors provided critical review and approved the version for publication.

## Conflict of interest

The authors declare that the research was conducted in the absence of any commercial or financial relationships that could be construed as a potential conflict of interest.

## Publisher's note

All claims expressed in this article are solely those of the authors and do not necessarily represent those of their affiliated organizations, or those of the publisher, the editors and the reviewers. Any product that may be evaluated in this article, or claim that may be made by its manufacturer, is not guaranteed or endorsed by the publisher.

## Abbreviations

AI: artificial intelligence
CI: confidence interval
ICD-10, International Statistical Classification of Diseases and Related Health Problems: 10th revision
IOP: intraocular pressure
IRB: institutional review board.
