# Supplementary material for: Artificial intelligence approach for recommendation of pupil dilation test using medical interview and basic ophthalmologic examinations
Source: Front Med (Lausanne). 2022 Sep 13;9:967710. doi: 10.3389/fmed.2022.967710 (PMC9513048; doi:10.3389/fmed.2022.967710)
Supplement: Supplementary file 1 [file Data_Sheet_1.docx]

**Supplement 1. Input data format**

**1. Basic patient characteristics**

1) Age

2) Sex

3) Type and time of systemic disease (ICD-10 classification [version 2019]) and treatments (surgical / radiologic / chemical / other medical)

4) Type, time, and site of ophthalmologic disease (ICD-10 classification [version 2019]) and treatment (surgical / medical) (see also supplement 2)

5) Glasses / lens history

6) Familial history of ophthalmologic disease (ICD-10 classification [version 2019])

**2. Eye Symptom (evaluate each individual eye)**

1) Characteristics

| (1) Vision |  |  |  |
| --- | --- | --- | --- |
| Blurriness, general  (transient/continuous) | Blindness  (transient/continuous) | Field defect  (central/peripheral) | Double vision  (monocular/binocular) |
| Color abnormality | Night blindness | Daytime blindness | Afterimage |
| Distortion  (metamorphopsia) | Different sizes  (aniseikonia) | Floaters | Fixed spot  (single/multiple) |
| Flash | Halo around light | Starburst around light | Poor near vision |
| (2) Pain |  |  |  |
| Location of eye  (in, behind, around eye, eyelid) | Aspect  (sharp, dull, pulsatile, or aggressive) |  |  |
| (3) Other sense |  |  |  |
| Burning sense | Dryness | Foreign body sense | Irritation or stinging |
| Itchiness | Strain or fatigue |  |  |
| (4) Other symptoms |  |  |  |
| a. Eyelid or Orbit |  |  |  |
| Bump or lump  (diffuse or focal) | Crusty | Redness (rash) | Pigment, eyelid  (diffuse or focal) |
| Vesicle or pustule |  |  |  |
| Spasm or twitching | Drooping (ptosis) | Lagophthalmos | Asymmetric eye size |
| Turn out (ectropion) | Turn in (entropion) | Eyelash problems | Tearing eyelid |
| b. Eyeball |  |  |  |
| Mass, conjunctiva | Pigment, conjunctiva | Swelling, conjunctiva | Other conjunctiva |
| Mass, cornea | Pigment, cornea | Other cornea | Other eye symptom |
| Red eye  (diffuse or focal) | Discharge | Teardrop  (spontaneous/windy/asymmetric) | Light sensitivity  (photophobia) |
| Dilated Pupil  (mydriasis) | Different pupil size  (anisocoria) | Small pupil  (miosis) | Different iris color |
| Crossed eye or squinting | Jumping or thrilling | Exophthalmos | Enophthalmos |
| d. Neurologics |  |  |  |
| Headache | Dizziness | Nausea/Vomiting | Facial Palsy (Focal/Hemi/Total) |

2) Duration

3) Time aspect

| 1) Continuity: lasting more than 24 hours |
| --- |
| 2) Progress: improve/constant/fluctuate/aggravate |

4) Events (also including patient subjective related event)

| Sharp/Penetrating | Blunt trauma | Chemical | Heat or Cold |
| --- | --- | --- | --- |
| Arc | Solar | Foreign body | Contact lens |
| Eye disease | Eye surgery or  procedure | Systemic disease | Systemic treatment |
| Rubbing | Other physical event |  |  |

5) Check-up or screening

| Refractive surgery  (i.e. LASIK, LASEK, phakic intraocular lens) | Screening for cornea (i.e. endothelial cell count, familial history of corneal dystrophy or keratoconus) | Screening for cataract or other lenticula disease | Increased intraocular pressure/Glaucoma suspect |
| --- | --- | --- | --- |
| Screening for chorioretinal disease (i.e. DMR, ARMD) | Screening for strabismus | Screening for orbital periorbital lesions (i.e. herpes zoster) | Plastic surgery |
| Screening for brain lesion or surgery (i.e. brain tumor, hematoma, and IICP) | Screening for other head and neck disease (i.e. facial palsy, sinusitis, and malignancies) | Screening for systemic disease and treatment including metabolic/immune disease/medication | Screening for congenital anomalies |

**3. Basic ophthalmologic examinations**

1) Vision (visual acuity, decimal-scaled)

(1) Uncorrected distance visual acuity

(2) Corrected distance visual acuity (with glasses or lens), if possible

(3) Corrected distance visual acuity with autokeratometry

2) Intraocular pressure measurement with non-contact tonometry

3) Autokeratometry

(1) Refractive errors (sphere, cylinder, and cylindrical axis)

(2) Keratometric values (flatter corneal power, steeper corneal power, axis of steeper corneal power)
