# Supplementary material for: Artificial intelligence approach for recommendation of pupil dilation test using medical interview and basic ophthalmologic examinations
Source: Front Med (Lausanne). 2022 Sep 13;9:967710. doi: 10.3389/fmed.2022.967710 (PMC9513048; doi:10.3389/fmed.2022.967710)
Supplement: Supplementary file 2 [file Data_Sheet_2.docx]

**Supplement 2. International Statistical Classification of Diseases and Related Health Problems 10th Revision (ICD-10) Version for 2010: Chapter VII Diseases of the eye and adnexa (H00-H59)**

**1. This chapter contains the following blocks:**

| H00-H06 | Disorders of eyelid, lacrimal system and orbit |
| --- | --- |
| H10-H13 | Disorders of conjunctiva |
| H15-H22 | Disorders of sclera, cornea, iris and ciliary body |
| H25-H28 | Disorders of lens  ("Y" when symptom and vision decrement caused by the disorders of lens) |
| H30-H36 | Disorders of choroid and retina  ("N" when small inactive peripheral chorioretinal scars in H31.0, mild hypertensive retinopathy in H35.0, asymptomatic small drusen in H35.3, and no risky lesions for rhegmatogenous retinal detachment in H35.4)^18- 20^ |
| H40-H42 | Glaucoma |
| H43-H45 | Disorders of vitreous body and globe |
| H46-H48 | Disorders of optic nerve and visual pathways  ("Y" when H46, H47.0, H48.0 with optic disc lesion, and H47.1-3) |
| H49-H52 | Disorders of ocular muscles, binocular movement, accommodation and refraction |
| H53-H54 | Visual disturbances and blindness |
| H55-H59 | Other disorders of eye and adnexa |

**2. This chapter does not contain in detail the following blocks: (Combined diagnosis with the eye and adnexa disease)**

| Certain conditions originating in the perinatal period (P00-P96) |
| --- |
| Certain infectious and parasitic diseases (A00-B99) |
| Complications of pregnancy, childbirth and the puerperium (O00-O99) |
| Congenital malformations, deformations and chromosomal abnormalities (Q00-Q99) |
| Endocrine, nutritional and metabolic diseases (E00-E90) |
| Injury, poisoning and certain other consequences of external causes (S00-T98) |
| Neoplasms (C00-D48) |
| Symptoms, signs and abnormal clinical and laboratory findings, not elsewhere classified (R00-R99) |

**3. Asterisk categories for this chapter are provided as follows:**

| H03* | Disorders of eyelid in diseases classified elsewhere |
| --- | --- |
| H06* | Disorders of lacrimal system and orbit in diseases classified elsewhere |
| H13* | Disorders of conjunctiva in diseases classified elsewhere |
| H19* | Disorders of sclera and cornea in diseases classified elsewhere |
| H22* | Disorders of iris and ciliary body in diseases classified elsewhere |
| H28* | Cataract and other disorders of lens in diseases classified elsewhere |
| H32* | Chorioretinal disorders in diseases classified elsewhere |
| H36* | Retinal disorders in diseases classified elsewhere |
| H42* | Glaucoma in diseases classified elsewhere |
| H45* | Disorders of vitreous body and globe in diseases classified elsewhere |
| H48* | Disorders of optic [2nd] nerve and visual pathways in diseases classified elsewhere |
| H58* | Other disorders of eye and adnexa in diseases classified elsewhere |

**4. Details**

**1) Detail: Disorders of eyelid, lacrimal system and orbit (H00-H06)**

| H00 | Disorders of eyelid, lacrimal system and orbit |
| --- | --- |
| H00.0 | Hordeolum and other deep inflammation of eyelid  (Abscess, furuncle, or stye of eyelid) |
| H00.1 | Chalazion |

| H01 | Other inflammation of eyelid |
| --- | --- |
| H01.0 | Blepharitis  (Exclusion: blepharoconjunctivitis [H10.5]) |
| H01.1 | Noninfectious dermatoses of eyelid  (Allergic/contact/eczematous dermatitis, Discoid lupus erythematosus, or Xeroderma of eyelid) |
| H01.8 | Other specified inflammation of eyelid |
| H01.9 | Inflammation of eyelid, unspecified |

| H02 | Other disorders of eyelid |
| --- | --- |
| H02.0 | Entropion and trichiasis of eyelid |
| H02.1 | Ectropion of eyelid |
| H02.2 | Lagophthalmos |
| H02.3 | Blepharochalasis |
| H02.4 | Ptosis of eyelid |
| H02.5 | Other disorders affecting eyelid function  (Ankyloblepharon, Blepharophimosis, or Lid retraction)  (Exclusion: blepharospasm [G24.5], tic (disorder) NOS [F95.9], psychogenic [F95.-], or organic origin [G25.6]) |
| H02.6 | Xanthelasma of eyelid |
| H02.7 | Other degenerative disorders of eyelid and periocular area  (Chloasma, madarosis, or vitiligo of eyelid) |
| H02.8 | Other specified disorders of eyelid  (Hypertrichosis of eyelid, or retained foreign body in eyelid) |

| H03* | Disorders of eyelid in diseases classified elsewhere |
| --- | --- |
| H03.0* | Parasitic infestation of eyelid in diseases classified elsewhere  (Dermatitis of eyelid due to Demodex species [B88.0])  (Parasitic infestation of eyelid in: leishmaniasis [B55.-], loiasis [B74.3], onchocerciasis [B73], or phthiriasis [B85.3]) |
| H03.1* | Involvement of eyelid in other infectious diseases classified elsewhere  (Involvement of eyelid in: herpesviral [herpes simplex] infection [B00.5+], leprosy [A30.-], molluscum contagiosum [B08.1], tuberculosis [A18.4], yaws [A66.-], or zoster [B02.3]) |
| H03.8* | Involvement of eyelid in other diseases classified elsewhere  (Involvement of eyelid in impetigo [L01.0]) |

| H04 | Disorders of lacrimal system  (Exclusion: congenital malformations of lacrimal system [Q10.4-Q10.6]) |
| --- | --- |
| H04.0 | Dacryoadenitis (Chronic enlargement of lacrimal gland) |
| H04.1 | Other disorders of lacrimal gland  (Dacryops, dry eye syndrome, lacrimal cyst, lacrimal gland atrophy) |
| H04.2 | Epiphora |
| H04.3 | Acute and unspecified inflammation of lacrimal passages  (Acute, subacute or unspecified phase of dacryocystitis (phlegmonous), dacryopericystitis, or lacrimal canaliculitis)  (Exclusion: neonatal dacryocystitis [P39.1]) |
| H04.4 | Chronic inflammation of lacrimal passages  (Chronic phase of dacryocystitis, lacrimal canaliculitis, or lacirmal mucocele) |
| H04.5 | Stenosis and insufficiency of lacrimal passages  (Dacryolith, eversion of lacrimal punctum, or stenosis of lacrimal (canaliculi, duct, or sac)) |
| H04.6 | Other changes in lacrimal passages (lacrimal fistula |
| H04.8 | Other disorders of lacrimal system |
| H04.9 | Disorder of lacrimal system, unspecified |

| H05 | Disorders of orbit  (Exclusion: congenital malformation of orbit [Q10.7]) |
| --- | --- |
| H05.0 | Acute inflammation of orbit  (Abscess, cellulitis, osteomyelitis, or periostitis of orbit, and tenonitis) |
| H05.1 | Chronic inflammatory disorders of orbit (Granuloma of orbit) |
| H05.2 | Exophthalmic conditions  (Displacement of globe (lateral) NOS, and haemorrhage or oedema of orbit) |
| H05.3 | Deformity of orbit (atrophy, or exostosis of orbit) |
| H05.4 | Enophthalmos |
| H05.5 | Retained (old) foreign body following penetrating wound of orbit  (Retrobulbar foreign body) |
| H05.8 | Other disorders of orbit (Cyst of orbit) |
| H05.9 | Disorder of orbit, unspecified |

| H06* | Disorders of lacrimal system and orbit in diseases classified elsewhere |
| --- | --- |
| H06.0* | Disorders of lacrimal system in diseases classified elsewhere |
| H06.1* | Parasitic infestation of orbit in diseases classified elsewhere  (Echinococcus infection of orbit [B67.-], or Myiasis of orbit [B87.2]) |
| H06.2* | Dysthyroid exophthalmos (E05.-) |
| H06.3* | Other disorders of orbit in diseases classified elsewhere |

**2) Detail: Disorders of conjunctiva (H10-H13)**

| H10 | Conjunctivitis  (Exclusion: keratoconjunctivitis [H16.2]) |
| --- | --- |
| H10.0 | Mucopurulent conjunctivitis |
| H10.1 | Acute atopic conjunctivitis |
| H10.2 | Other acute conjunctivitis |
| H10.3 | Acute conjunctivitis, unspecified  (Exclusion: ophthalmia neonatorum NOS [P39.1]) |
| H10.4 | Chronic conjunctivitis |
| H10.5 | Blepharoconjunctivitis |
| H10.8 | Other conjunctivitis |
| H10.9 | Conjunctivitis, unspecified |

| H11 | Other disorders of conjunctiva  (Exclusion: keratoconjunctivitis [H16.2]) |
| --- | --- |
| H11.0 | Pterygium  (Exclusion: pseudopterygium [H11.8]) |
| H11.1 | Conjunctival degenerations and deposits  (Conjunctival argyrosis, concretions, pigmentation, or xerosis NOS) |
| H11.2 | Conjunctival scars (symblepharon) |
| H11.3 | Conjunctival haemorrhage (subconjunctival haemorrhage) |
| H11.4 | Other conjunctival vascular disorders and cysts  (Conjunctival aneurysm, hyperaemia, or oedema) |
| H11.8 | Other specified disorders of conjunctiva (Pseudopterygium) |
| H11.9 | Disorder of conjunctiva, unspecified |

| H13* | Disorders of conjunctiva in diseases classified elsewhere |
| --- | --- |
| H13.0* | Filarial infection of conjunctiva [B74.-] |
| H13.1* | Conjunctivitis in infectious and parasitic diseases classified elsewhere  (Conjunctivitis due to acanthamoeba [B60.1], adenoviral follicular (acute) [B30.1], chlamydial [A74.0], diphtheritic [A36.8], gonococcal [A54.3], haemorrhagic (acute, epidemic) [B30.3], herpesviral (herpes simplex) [B00.5], meningococcal [A39.8], Newcastle [B30.8], or zoster [B02.3]) |
| H13.2* | Conjunctivitis in other diseases classified elsewhere |
| H13.3* | Ocular pemphigoid [L12.-] |
| H13.8* | Other disorders of conjunctiva in diseases classified elsewhere |

**3) Detail: Disorders of sclera, cornea, iris and ciliary body (H15-H22)**

| H15 | Disorders of sclera |
| --- | --- |
| H15.0 | Scleritis |
| H15.1 | Episcleritis |
| H15.8 | Other disorders of sclera (equatorial staphyloma, or scleral ectasia)  (Exclusion: degenerative myopia [H44.2]) |
| H15.9 | Disorder of sclera, unspecified |

| H16 | Keratitis |
| --- | --- |
| H16.0 | Corneal ulcer  (Corneal ulcer NOS, central, marginal, perforated, ring, or with hypopyon)  (Mooren’s ulcer) |
| H16.1 | Other superficial keratitis without conjunctivitis  (Keratitis: areolar, filamentary, nummular, stellate, striate, or superficial punctate)  (Photokeratitis, snow blindness) |
| H16.2 | Keratoconjunctivitis  (Keratoconjunctivitis NOS, exposure, neurotrophic, or phlyctenular)  (Ophthalmia nodosa, superficial keratitis with conjunctivitis) |
| H16.3 | Interstitial and deep keratitis |
| H16.4 | Corneal neovascularization (corneal ghost vessels, or pannus) |
| H16.8 | Other keratitis |
| H16.9 | Keratitis, unspecified |

| H17 | Corneal scars and opacities |
| --- | --- |
| H17.0 | Adherent leukoma |
| H17.1 | Other central corneal opacity |
| H17.8 | Other corneal scars and opacities |
| H17.9 | Corneal scar and opacity, unspecified |

| H18 | Other disorders of cornea |
| --- | --- |
| H18.0 | Corneal pigmentations and deposits  (Haematocornea, Kayser-Fleischer ring, Krukenberg spindle, Staehli line)  (Use additional external cause code (Chapter XX), if desired, to identify drug, if drug-induced) |
| H18.1 | Bullous keratopathy  (Exclusion: keratopathy (bullous aphakic) following cataract surgery [H59.0]) |
| H18.2 | Other corneal oedema |
| H18.3 | Changes in corneal membranes (fold, or rupture in Descemet membrane) |
| H18.4 | Corneal degeneration (arcus senilis, or band keratopathy)  (Exclusion: Mooren’s ulcer [H16.0]) |
| H18.5 | Hereditary corneal dystrophies  (Epithelial, granular, lattice, or macular corneal dystrophy, and Fuchs dystrophy) |
| H18.6 | Keratoconus |
| H18.7 | Other corneal deformities  (Corneal ectasia, or staphyloma, and descemetocele)  (Exclusion: congenital malformations of cornea [Q13.3-Q13.4]) |
| H18.8 | Other specified disorders of cornea  (Anaesthesia, hypaesthesia, or recurrent erosion of cornea) |
| H18.9 | Disorder of cornea, unspecified |

| H19* | Disorders of sclera and cornea in diseases classified elsewhere |
| --- | --- |
| H19.0* | Scleritis and episcleritis in diseases classified elsewhere  (Syphilitic episcleritis [A52.7], tuberculous episcleritis [A18.5], or zoster scleritis [B02.3]) |
| H19.1* | Herpesviral keratitis and keratoconjunctivitis [B00.5] (Dendritic and disciform keratitis) |
| H19.2* | Keratitis and keratoconjunctivitis in other infectious and parasitic diseases classified elsewhere  (Epidemic keratoconjunctivitis [B30.0])  (Keratitis and keratoconjunctivitis (interstitial) in acanthamoebiasis [B60.1], measles [B05.8], syphilis [A50.3], tuberculosis [A18.5], or zoster [B02.3]) |
| H19.8* | Keratitis and keratoconjunctivitis in other diseases classified elsewhere  (Keratoconjunctivitis sicca [M35.0]) |
| H19.9* | Other disorders of sclera and cornea in diseases classified elsewhere  (Keratoconus in Down syndrome [Q90.-]) |

| H20 | Iridocyclitis |
| --- | --- |
| H20.0 | Acute and subacute iridocyclitis  (Acute, recurrent, or subacute phase of anterior uveitis, cyclitis, or iritis) |
| H20.1 | Chronic iridocyclitis |
| H20.2 | Lens-induced iridocyclitis |
| H20.8 | Other iridocyclitis |
| H20.9 | Iridocyclitis, unspecified |

| H21 | Other disorders of iris and ciliary body  (Exclusion: sympathetic uveitis [H44.1]) |
| --- | --- |
| H21.0 | Hyphaema  (Exclusion: traumatic hyphaema [S05.1]) |
| H21.1 | Other vascular disorders of iris and ciliary body  (Neovascularization of iris or ciliary body, or rubeosis of iris) |
| H21.2 | Degeneration of iris and ciliary body  (Degeneration of iris (pigmentary) or pupillary margin)  (Iridoschisis, iris atrophy (essential, progressive), miotic pupillary cyst, or translucency of iris) |
| H21.3 | Cyst of iris, ciliary body and anterior chamber  (Cyst of iris, ciliary body or anterior chamber NOS, exudative, implantation, or parasitic)  (Exclusion: miotic pupillary cyst [H21.2]) |
| H21.4 | Pupillary membranes (iris bombé, or pupillary occlusion/seclusion) |
| H21.5 | Other adhesions and disruptions of iris and ciliary body  (Goniosynechiae, iridodialysis, chamber angle recession, synechiae (iris) NOS/anterior/posterior)  (Exclusion: corectopia [Q13.2]) |
| H21.8 | Other specified disorders of iris and ciliary body |
| H21.9 | Disorder of iris and ciliary body, unspecified |

| H22* | Disorders of iris and ciliary body in diseases classified elsewhere |
| --- | --- |
| H22.0* | Iridocyclitis in infectious and parasitic diseases classified elsewhere  (Iridocyclitis in gonococcal infection [A54.3], herpesviral (herpes simplex) infection [B00.5], syphilis (secondary) [A51.4], tuberculosis [A18.5], or zoster [B02.3]) |
| H22.1* | Iridocyclitis in other diseases classified elsewhere  (Iridocyclitis in ankylosing spondylitis [M45], or sarcoidosis [D86.8]) |
| H21.8* | Other disorders of iris and ciliary body in diseases classified elsewhere |

**4) Detail: Disorders of lens (H25-H28)**

| H25 | Senile cataract  (Exclusion: capsular glaucoma with pseudoexfoliation of lens [H40.1]) |
| --- | --- |
| H25.0 | Senile incipient cataract  (Coronary, cortical, or punctate senile cataract)  (Subcapsular polar senile cataract (anterior, or posterior), or Water clefts) |
| H25.1 | Senile nuclear cataract (cataracta brunescens, nuclear sclerosis cataract) |
| H25.2 | Senile cataract, morgagnian type (senile hypermature cataract) |
| H25.8 | Other senile cataract (combined forms of senile cataract) |
| H25.9 | Senile cataract, unspecified |

| H26 | Other cataract  (Exclusion: congenital cataract [Q12.0]) |
| --- | --- |
| H26.0 | Infantile, juvenile and presenile cataract |
| H26.1 | Traumatic cataract (use additional external cause code (Chapter XX), if desired, to identify cause) |
| H26.2 | Complicated cataract  (Cataract in chronic iridocyclitis, cataract secondary to ocular disorders, or subcapsular glaucomatous flecks) |
| H26.3 | Drug-induced cataract (use additional external cause code (Chapter XX), if desired, to identify drug) |
| H26.4 | After-cataract (secondary cataract, or Soemmerring ring) |
| H26.8 | Other specified cataract |
| H26.9 | Cataract, unspecified |

| H27 | Other disorders of lens  (Exclusion: congenital lens malformations [Q12.-], mechanical complications of intraocular lens [T85.2], or pseudophakia [Z96.1]) |
| --- | --- |
| H27.0 | Aphakia |
| H27.1 | Dislocation of lens |
| H27.8 | Other specified disorders of lens |
| H27.9 | Disorder of lens, unspecified |

| H28* | Cataract and other disorders of lens in diseases classified elsewhere |
| --- | --- |
| H28.0* | Diabetic cataract (E10-E14 with common fourth character .3) |
| H28.1* | Cataract in other endocrine, nutritional and metabolic diseases  (Cataract in hypoparathyroidism [E20.-], or malnutrition-dehydration cataract [E40-E46]) |
| H28.2* | Cataract in other diseases classified elsewhere (Myotonic cataract [G71.1]) |
| H28.8* | Other disorders of lens in diseases classified elsewhere |

**5) Detail: Disorders of choroid and retina (H30-H36)**

| H30 | Chorioretinal inflammation |
| --- | --- |
| H30.0 | Focal chorioretinal inflammation  (Focal chorioretinitis, choroiditis, retinitis, or retinochoroiditis) |
| H30.1 | Disseminated chorioretinal inflammation  (Disseminated chorioretinitis, choroiditis, retinitis, or retinochoroiditis)  (Exclusion: exudative retinopathy [H35.0]) |
| H30.2 | Posterior cyclitis (pars planitis) |
| H30.8 | Other chorioretinal inflammations (Harada disease) |
| H30.9 | Chorioretinal inflammation, unspecified  (Chorioretinitis, choroiditis, retinitis, or retinochoroiditis NOS) |

| H31 | Other disorders of choroid |
| --- | --- |
| H31.0 | Chorioretinal scars  (Macula scars of posterior pole (postinflammatory, or post-traumatic), solar retinopathy) |
| H31.1 | Choroidal degeneration (atrophy, or sclerosis of choroid)  (Exclusion: angioid streaks [H35.3]) |
| H31.2 | Hereditary choroidal dystrophy  (Choroideremia, dystrophy (central areolar, generalized, or peripapillary), or gyrate atrophy)  (Exclusion: ornithinaemia [E72.4]) |
| H31.3 | Choroidal haemorrhage and rupture (NOS, or expulsive) |
| H31.4 | Choroidal detachment |
| H31.8 | Other specified disorders of choroid (choroidal neovascularization) |
| H31.9 | Disorder of choroid, unspecified |

| H32* | Chorioretinal disorders in diseases classified elsewhere |
| --- | --- |
| H32.0* | Chorioretinal inflammation in infectious and parasitic diseases classified elsewhere  (Chorioretinitis in late syphilitic [A52.7], toxoplasma [B58.0], or tuberculous [A18.5]) |
| H32.8* | Other chorioretinal disorders in diseases classified elsewhere  (Albuminuric retinitis [N18.5], or renal retinitis [N18.5]) |

| H33 | Retinal detachments and breaks  (Exclusion: detachment of retinal pigment epithelium [H35.7]) |
| --- | --- |
| H33.0 | Retinal detachment with retinal break (Rhegmatogenous retinal detachment) |
| H33.1 | Retinoschisis and retinal cysts  (Cyst of ora serrata, parasitic cyst of retina NOS, or pseudocyst of retina)  (Exclusion: congenital retinoschisis [Q14.1], or microcystoid degeneration of retina [H35.4]) |
| H33.2 | Serous retinal detachment (Retinal detachment NOS or without retinal break)  (Exclusion: central serous chorioretinopathy [H35.7]) |
| H33.3 | Retinal breaks without detachment  (Horseshoe tear, or round hole of retina, without detachment)  (Operculum, or retinal break NOS)  (Exclusion: chorioretinal scars after surgery for detachment [H59.8], or peripheral retinal degeneration without break [H35.4]) |
| H33.4 | Traction detachment of retina (proliferative vitreo-retinopathy with retinal detachment) |
| H33.5 | Other retinal detachments |

| H34 | Retinal vascular occlusions  (Exclusion: amaurosis fugax [G45.3]) |
| --- | --- |
| H34.0 | Transient retinal artery occlusion |
| H34.1 | Central retinal artery occlusion |
| H34.2 | Other retinal artery occlusions  (Hollenhorst plaque, branch/partial retinal artery occlusion, or retinal microembolism) |
| H34.8 | Other retinal vascular occlusions  (Retinal vein occlusion: central, incipient, partial, or tributary) |
| H34.9 | Retinal vascular occlusion, unspecified |

| H35 | Other retinal disorders |
| --- | --- |
| H35.0 | Background retinopathy and retinal vascular changes (Changes in retinal vascular appearance)  (Retinal micro-aneurysms, neovascularization, perivasculitis, varices, vascular sheathing, or vasculitis)  (Retinopathy NOS, background NOS, Coats, exudative, or hypertensive) |
| H35.1 | Retinopathy of prematurity (retrolental fibroplasia) |
| H35.2 | Other proliferative retinopathy (proliferative vitreo-retinopathy)  (Exclusion: proliferative vitreo-retinopathy with retinal detachment [H33.4]) |
| H35.3 | Degeneration of macula and posterior pole  (Angioid streaks, cyst, drusen (degenerative), hole, or puckering of macula)  (Kuhnt-Junius degeneration, senile macular degeneration (atrophic, exudative), toxic maculopathy (use additional external cause code (Chapter XX), if desired, to identify drug, if drug-induced) |
| H35.4 | Peripheral retinal degeneration  (Degeneration NOS, lattice, microcystoid, palisade, paving stone, or reticular)  (Exclusion: with retinal break [H33.3]) |
| H35.5 | Hereditary retinal dystrophy  (Retinal (albipunctate, pigmentary, or vitelliform), tapetoretinal, or vitreoretinal dystrophy)  (Retinitis pigmentosa, or Stargardt disease) |
| H35.6 | Retinal haemorrhage |
| H35.7 | Separation of retinal layers  (Central serous chorioretinopathy, or detachment of retinal pigment epithelium) |
| H35.8 | Other specified retinal disorders |
| H35.9 | Retinal disorder, unspecified |

| H36* | Retinal disorders in diseases classified elsewhere |
| --- | --- |
| H36.0* | Diabetic retinopathy (E10-E14 with common fourth character .3) |
| H36.8* | Other retinal disorders in diseases classified elsewhere  (Atherosclerotic retinopathy [I70.8], proliferative sickle-cell retinopathy [D57.-], or retinal dystrophy in lipid storage disorders [E75.-]) |

**6) Detail: Glaucoma (H40-H42)**

| H40 | Glaucoma  (Exclusion: absolute glaucoma [H44.5], congenital glaucoma [Q15.0], or traumatic glaucoma due to birth injury [P15.3]) |
| --- | --- |
| H40.0 | Glaucoma suspect (ocular hypertension) |
| H40.1 | Primary open-angle glaucoma  (Capsular with pseudoexfoliation of lens, chronic simple, low-tension, or pigmentary glaucoma) |
| H40.2 | Primary angle-closure glaucoma  (Acute, intermittent, or chronic angle-closure glaucoma) |
| H40.3 | Glaucoma secondary to eye trauma (use additional code, if desired, to identify cause) |
| H40.4 | Glaucoma secondary to eye inflammation (use additional code, if desired, to identify cause) |
| H40.5 | Glaucoma secondary to other eye disorders (use additional code, if desired, to identify cause) |
| H40.6 | Glaucoma secondary to drugs (use additional external cause code (Chapter XX), if desired, to identify drug) |
| H35.8 | Other glaucoma |
| H35.9 | Glaucoma, unspecified |

| H42* | Glaucoma in diseases classified elsewhere |
| --- | --- |
| H42.0* | Glaucoma in endocrine, nutritional and metabolic diseases  (Glaucoma in amyloidosis [E85.-], or Lowe syndrome [E72.0]) |
| H42.8* | Glaucoma in other diseases classified elsewhere  (Glaucoma in onchocerciasis [B73]) |

**7) Detail: Disorders of vitreous body and globe (H43-H45)**

| H43 | Disorders of vitreous body |
| --- | --- |
| H43.0 | Vitreous prolapse  (Exclusion: vitreous syndrome following cataract surgery [H59.0]) |
| H43.1 | Vitreous haemorrhage |
| H43.2 | Crystalline deposits in vitreous body |
| H43.3 | Other vitreous opacities (vitreous membranes and strands) |
| H43.8 | Other disorders of vitreous body (vitreous degeneration, or detachment)  (Exclusion: proliferative vitreo-retinopathy with retinal detachment [H33.4]) |
| H43.9 | Disorder of vitreous body, unspecified |

| H44 | Disorders of globe (disorders affecting multiple structures of eye) |
| --- | --- |
| H44.0 | Purulent endophthalmitis (panophthalmitis, or vitreous abscess) |
| H44.1 | Other endophthalmitis (parasitic endophthalmitis NOS, or sympathetic uveitis) |
| H44.2 | Degenerative myopia |
| H44.3 | Other degenerative disorders of globe (chalcosis, or siderosis of eye) |
| H44.4 | Hypotony of eye |
| H44.5 | Degenerated conditions of globe (absolute glaucoma, atrophy of globe, or phthisis bulbi) |
| H44.6 | Retained (old) intraocular foreign body, magnetic |
| H44.7 | Retained (old) intraocular foreign body, nonmagnetic |
| H43.8 | Other disorders of globe (haemophthalmos, or luxation of globe) |
| H43.9 | Disorder of globe, unspecified |

| H45* | Disorders of vitreous body and globe in diseases classified elsewhere |
| --- | --- |
| H45.0* | Vitreous haemorrhage in diseases classified elsewhere |
| H45.1* | Endophthalmitis in diseases classified elsewhere  (Endophthalmitis in cysticercosis [B69.1], onchocerciasis [B73], or toxocariasis [B83.0]) |
| H45.8* | Other disorders of vitreous body and globe in diseases classified elsewhere |

**8) Detail: Disorders of optic nerve and visual pathways (H46-H48)**

| H46 | Optic neuritis  (Inclusion: optic neuropathy except ischaemic, papillitis, or retrobulbar neuritis NOS)  (Exclusion: ischaemic optic neuropathy [H47.0], or neuromyelitis optica [G36.0]) |
| --- | --- |

| H47 | Other disorders of optic [2nd] nerve and visual pathways |
| --- | --- |
| H47.0 | Disorders of optic nerve, not elsewhere classified  (Compression of optic nerve, Kennedy syndrome, haemorrhagic in optic nerve sheath, or ischaemic optic neuropathy) |
| H47.1 | Papilloedema, unspecified |
| H47.2 | Optic atrophy (temporal pallor of optic disc) |
| H47.3 | Other disorders of optic disc  (Drusen of optic disc, or pseudopapilloedema) |
| H47.4 | Disorders of optic chiasm |
| H47.5 | Disorders of other visual pathways  (Disorders of optic tracts, geniculate nuclei and optic radiations) |
| H47.6 | Disorders of visual cortex |
| H47.7 | Disorder of visual pathways, unspecified |

| H48* | Disorders of optic [2nd] nerve and visual pathways in diseases classified elsewhere |
| --- | --- |
| H48.0* | Optic atrophy in diseases classified elsewhere  (Optic atrophy in late syphilis [A52.1]) |
| H48.1* | Retrobulbar neuritis in diseases classified elsewhere  (Retrobulbar neuritis in late syphilis [A52.1], meningococcal infection [A39.8], or multiple sclerosis [G35]) |
| H48.8* | Other disorders of optic nerve and visual pathways in diseases classified elsewhere |

**9) Detail: Disorders of ocular muscles, binocular movement, accommodation and refraction**

**(H49-H52)**

| H49 | Paralytic strabismus  (Exclusion: ophthalmoplegia internal [H52.5], internuclear [H51.2], progressive supranuclear [G23.1]) |
| --- | --- |
| H49.0 | Third [oculomotor] nerve palsy |
| H49.1 | Fourth [trochlear] nerve palsy |
| H49.2 | Sixth [abducent] nerve palsy |
| H49.3 | Total (external) ophthalmoplegia |
| H49.4 | Progressive external ophthalmoplegia |
| H49.8 | Other paralytic strabismus  (External ophthalmoplegia NOS, or Kearns-Sayre syndrome) |
| H49.9 | Paralytic strabismus, unspecified |

| H50 | Other strabismus |
| --- | --- |
| H50.0 | Convergent concomitant strabismus  (Esotropia (alternating, monocular), except intermittent) |
| H50.1 | Divergent concomitant strabismus  (Exotropia (alternating, monocular), except intermittent) |
| H50.2 | Vertical strabismus (hypertropia, or hypotropia) |
| H50.3 | Intermittent heterotropia (esotropia, or exotropia) |
| H50.4 | Other and unspecified heterotropia  (Concomitant strabismus NOS, cyclotropia, microtropia, or monofixation syndrome) |
| H50.5 | Heterophoria  (Alternating hyperphoria, esophoria, or exophoria) |
| H50.6 | Mechanical strabismus  (Brown sheath syndrome, strabismus due to adhesions, or traumatic limitation of duction of eye muscle) |
| H50.8 | Other specified strabismus  (Duane syndrome) |
| H50.9 | Strabismus, unspecified |

| H51 | Other disorders of binocular movement |
| --- | --- |
| H51.0 | Palsy of conjugate gaze |
| H51.1 | Convergence insufficiency and excess |
| H51.2 | Internuclear ophthalmoplegia |
| H51.8 | Other specified disorders of binocular movement |
| H51.9 | Disorder of binocular movement, unspecified |

| H52 | Disorders of refraction and accommodation |
| --- | --- |
| H52.0 | Hypermetropia |
| H52.1 | Myopia  (Exclusion: degenerative myopia [H44.2]) |
| H52.2 | Astigmatism |
| H52.3 | Anisometropia and aniseikonia |
| H52.4 | Presbyopia |
| H52.5 | Disorders of accommodation  (Internal ophthalmoplegia (complete, total))  (Paresis, or spasm of accommodation) |
| H52.6 | Other disorders of refraction |
| H52.7 | Disorder of refraction, unspecified |

**10) Detail: Visual disturbances and blindness (H53-H54)**

| H53 | Visual disturbances |
| --- | --- |
| H53.0 | Amblyopia ex anopsia (anisometropic, deprivation, or strabismic) |
| H53.1 | Subjective visual disturbances (Asthenopia, day blindness, hemeralopia, metamorphopsia, photophobia, scintillating scotoma, sudden visual loss, or visual halos)  (Exclusion: visual hallucinations [R44.1]) |
| H53.2 | Diplopia (double vision) |
| H53.3 | Other disorders of binocular vision (abnormal retinal correspondence, fusion with defective stereopsis, simultaneous visual perception without fusion, or suppression of binocular vision) |
| H53.4 | Visual field defects (enlarged blind spot, generalized contraction of visual field, hemianopsia, quadrant anopsia, arcuate/Bjerrum/central/ring scotoma) |
| H53.5 | Colour vision deficiencies (Achromatopsia, acquired colour vision deficiency, colour blindness, deuteranomaly, deuteranopia, protanomaly, protanopia, tritanomaly, or tritanopia)  (Exclusion: day blindness [H53.1]) |
| H53.6 | Night blindness  (Exclusion: vitamin A deficiency [E50.5]) |
| H53.8 | Other visual disturbances |
| H53.9 | Visual disturbance, unspecified |

| H54 | Visual impairment including blindness (binocular or monocular)  Note: For definition of visual impairment categories see table below.  (Exclusion: amaurosis fugax [G45.3]) |
| --- | --- |
| H54.0 | Blindness, binocular (Visual impairment categories 3, 4, 5) |
| H54.1 | Severe visual impairment, binocular (Visual impairment category 2) |
| H54.2 | Moderate visual impairment, binocular (Visual impairment category 1) |
| H54.3 | Mild or no visual impairment, binocular (Visual impairment category 0) |
| H54.4 | Blindness, monocular (Visual impairment categories 3, 4, 5 in one eye and categories 0, 1, 2 or 9 in the other eye) |
| H54.5 | Severe visual impairment, monocular (Visual impairment category 2 in one eye and categories 0, 1 or 9 in other eye) |
| H54.6 | Moderate visual impairment, monocular (Visual impairment category 1 in one eye and categories 0 or 9 in other eye) |
| H54.9 | Unspecified visual impairment (binocular) (Visual impairment category 9) |

| Category | Presenting distance visual acuity | |
| --- | --- | --- |
|  | Worsen than: | Equal to or better than: |
| 0 Mild or no visual impairment |  | 6/18  3/10 (0.3)  20/70 |
| 1 Moderate visual impairment | 6/18  3/10 (0.3)  20/70 | 6/60  1/10 (0.1)  20/200 |
| 2 Severe visual impairment | 6/60  1/10 (0.1)  20/200 | 3/60  1/20 (0.05)  20/400 |
| 3 Blindness | 3/60  1/20 (0.05)  20/400 | 1/60*  1/50 (0.02)  5/300 (20/1200) |
| 4 Blindness | 1/60*  1/50 (0.02)  5/300 (20/1200) | Light perception |
| 5 Blindness | No light perception | |
| 9 | Undetermined or unspecified | |
|  | * or counts fingers (CF) at 1 metre. | |

**11) Detail: Other disorders of eye and adnexa (H55-H59)**

| H55 | Nystagmus and other irregular eye movements (NOS, congenital, deprivation, dissociated, or latent) |
| --- | --- |

| H57 | Other disorders of eye and adnexa |
| --- | --- |
| H57.0 | Anomalies of pupillary function |
| H57.1 | Ocular pain |
| H57.8 | Other specified disorders of eye and adnexa |
| H57.9 | Disorder of eye and adnexa, unspecified |

| H58* | Other disorders of eye and adnexa in diseases classified elsewhere |
| --- | --- |
| H58.0* | Anomalies of pupillary function in diseases classified elsewhere  (Argyll Robertson phenomenon or pupil, syphilitic [A52.1]) |
| H58.1* | Visual disturbances in diseases classified elsewhere |
| H58.8* | Other specified disorders of eye and adnexa in diseases classified elsewhere  (Syphilitic oculopathy NEC; congenital early [A50.0]/late [A50.3], early (secondary) [A51.4], or late [A52.7])  (Thyroid (thyrotoxic) eye disease [E05.-]) |

| H59 | Postprocedural disorders of eye and adnexa, not elsewhere classified  (Exclusion: mechanical complication of intraocular lens [T85.2], or other ocular prosthetic devices, implants and grafts [T85.3], and pseudophakia [Z96.1]) |
| --- | --- |
| H59.0 | Keratopathy (bullous aphakic) following cataract surgery  (Vitreous (touch) syndrome, or vitreal corneal syndrome) |
| H59.1 | Other postprocedural disorders of eye and adnexa  (Bleb-associated endophthalmitis, chorioretinal scars after surgery for detachment, |
| H59.8 | inflammation (infection) of postprocedural bleb, or postprocedural blebitis) |
| H59.9 | Postprocedural disorder of eye and adnexa, unspecified |
